# A Pilot Study of Retail ‘Vape Shops’ in the San Francisco Bay Area

**DOI:** 10.18332/tpc/65229

**Published:** 2016-10-05

**Authors:** Andrea D Burbank, Johannes Thrul, Pamela M Ling

**Affiliations:** a University of California, San Francisco, United States

**Keywords:** Smoking cessation, e-cigarette, vape shops, Electronic Nicotine Delivery Systems (ENDS), tobacco retail environment

## Abstract

**INTRODUCTION:**

The use of electronic cigarettes or vape devices is increasing, and products are evolving rapidly. This study assessed retail vape shops in the San Francisco Bay Area to describe store characteristics, products offered, advertisements and health claims, as well as employees’ perceptions of their customers’ demographics, and practices to support smoking cessation.

**METHODS:**

We conducted store audits of shops that exclusively sell vape devices with physical addresses in San Francisco and Alameda counties (n=23, response rate 72%) and interviewed vape shop owners/employees.

**RESULTS:**

While all stores carried second and third generation vape devices, 83% of stores did not carry first generation devices. Employees estimated the majority of their customers bought devices for smoking cessation or to replace tobacco, and a small minority purchased for first-time recreational use. Employees most frequently recommended dosing nicotine based on usual cigarette consumption, adjusting doses based on “throat hit” or cravings, use of a second or third generation e-cigarette, and encouraged customers to experiment and customize to “whatever works for you” as smoking cessation advice.

**CONCLUSIONS:**

Vape shops report a significant number of their customers are interested in smoking cessation, and employees are giving smoking cessation advice. A subpopulation of customers includes some nicotine novices. Studies of vape shops should include both observations and interviews with employees in order to detect important informal practices that may differ from posted signs or printed advertising. These practices include cessation counseling, product claims, and custom discount prices or bargaining.

## INTRODUCTION

**Figure 1:**
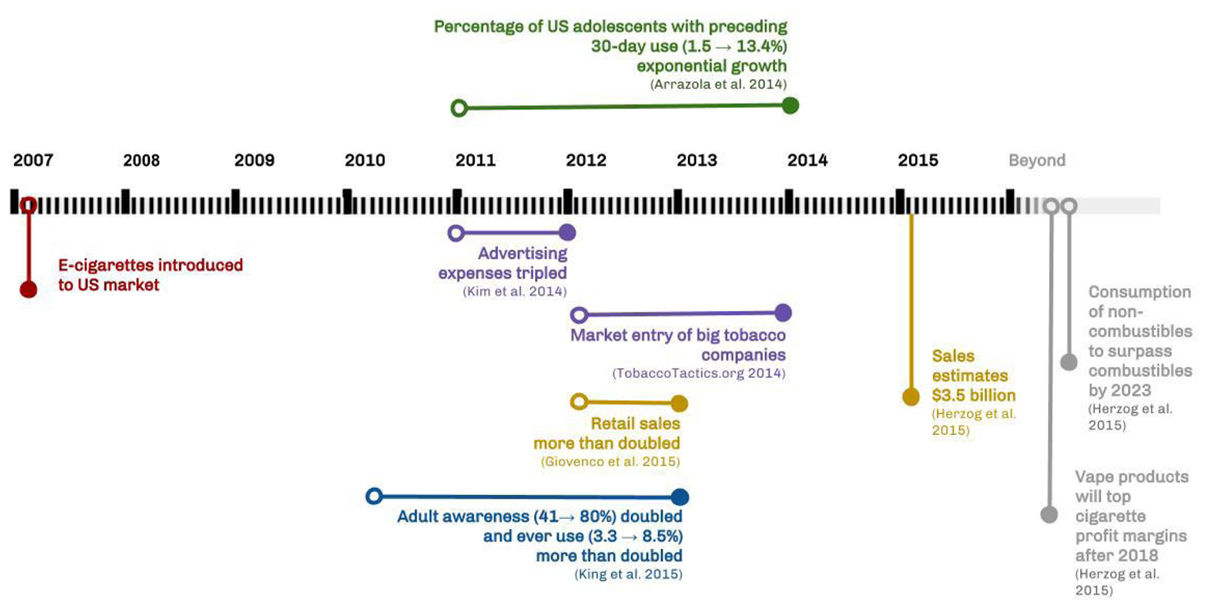
Integrated timeline for published estimates of retail market and public adoption of e-cigarettes 2007–2023^3,36–39,40^.

Electronic nicotine delivery systems (ENDS) (commonly called “electronic cigarettes”, “e-cigarettes” or “vapes”) are devices that aerosolize a flavored liquid (“e-juice”), usually containing nicotine for inhalation^1,2^. The ENDS industry has grown rapidly in recent years, with increases in sales and use by both adults and adolescents, with some industry analysts projecting that ENDS will eclipse combustible cigarettes in the future (Figure 1)^3^. ENDS have been estimated to be a $3.5 billion industry^4^ which was unregulated until the FDA extended its regulatory authority to include ENDS, effective August 8, 2016. Both ENDS devices and delivery systems have rapidly evolved, and over 460 brands of ENDS and >7,000 unique e-juice flavors were identified in 2014^5^. In addition, since 2007 ENDS have evolved into three distinct product lines. First generation devices (“cigalikes”) resemble combustible cigarettes, are commonly sold in convenience stores, and generally deliver less nicotine than a combustible cigarette^6,7^. Many first generation devices are disposable, and sell for less than $10. Second generation devices (“eGos” or “vape pens”) are larger than cigarettes, refillable, have easily assembled components, and are more frequently rechargeable rather than disposable^6^. The nicotine delivery is more similar to a combustible cigarette and the devices are commonly sold in kits^7^. Third generation devices (“mods”, “rebuildables”, or “advanced personal vaporizers”) come in a large array of customizable formats, generally including adjustable settings, stronger batteries or variable voltage for increased nicotine delivery, and refillable tanks for e-juice^6,7^. The proliferation of these second and third generation vape devices is driven by online sales and specialty vape shops^8,9^.

Most studies of ENDS marketing have analyzed brand websites,^5,10,11^ and less is known about the devices and e-juices for sale at vape shops, which are an increasingly important part of the ENDS market. Vape shops typically offer different ENDS products than those found in convenience stores, and seem likely to attract different consumers. A National Institutes of Health sponsored workshop in 2013 prioritized research on safety profiles of ENDS aerosol contents, physiologic effects, and “information on e-cigarette users”, “how the devices are used”, “identification of the best tools to assess these measures”, and “factors that drive use and influence patterns of use”^12^. Prior studies identified increasing numbers of vape shops in the USA,^8^ and described consumer perceptions of vape shops based on Yelp reviews^13^. A few studies have surveyed vape shop owners’ attitudes and beliefs about vaping and smoking cessation, messages to customers, and marketing practices,^14–16^ or conducted naturalistic observations of vape shop customers^17^. This previous research reported that vape shop owners generally believe ENDS to be a safe source of nicotine,^14,15^ and compare it to medical treatments^14^. Vape shops have been shown to use print and social media marketing, price discounts, specials, and loyalty programs to promote their products, as well as targeting specific groups including college students and long term smokers^16^. To expand the limited literature in this novel and rapidly changing research area,^9^ we undertook a pilot study of retail vape shops in the San Francisco Bay Area.

The study objective was to survey retail vape shops and vape shop employees in the San Francisco Bay Area to describe store characteristics, products and pricing of devices and e-juice, advertisements and health claims, vape shop employees’ perceptions of their customers’ demographics, and what, if any, smoking cessation advice employees provide.

## METHODS

### Procedures

We sampled shops exclusively selling ENDS and not other tobacco products with physical addresses in San Francisco and Alameda counties. Shops were identified using the online directory of businesses, Yelp, employing an established search strategy that has been shown to produce highly accurate search results^13,18^. We used the search terms “vape shop” or “vapor” paired with the location “Near: San Francisco, CA”. From Yelp we found 67 self-identified ‘vape shops’ in San Francisco or Alameda counties and conducted in-person visits between May 2015 and March 2016. A total of 35 stores were excluded (22 carried tobacco products, 12 were out of business, and 1 was whole sale only). Of the remaining 32 shops eligible for the study, 9 opted out and 23 completed assessments (response rate 72%).

### Engaging Vape Shop Employees

While tobacco retail assessments are frequently performed without engaging store employees,^19^ vape shop assessments required more engagement with employees. Simple price listings were often not available, as most ENDS sold in vape shops are customized for individual consumers, which affects the price of the device. Vape shop employees were knowledgeable about ENDS devices and e-juices, and expected to engage customers in conversation as part of the sales process, making observations without engagement difficult. During the time of this study, local ordinances restricting the sales and licensing of ENDS were being debated publicly, so vape shop employees were suspicious of authorities, including researchers. A casual, friendly and open-minded approach with prompt researcher identification and respectful request for permission to interview encouraged participation. Employees responded positively to reassurances that the researchers were not biased against vape shops, stressing the importance of neutral research, queries about what research the vape community would find valuable, offers to share study results with the vape shops, and assurances of anonymity. All of the shops participating in interviews gave contact emails to the research team to share study results.

### Measures

Data were recorded on modified versions of the UIC Vape Store Observation Form and the UIC Vape Store Merchant Interview Guide developed by Barker^20,21^. The measures assessed aspects of the store (e.g., location, hours and days of operation, employees, amenities), customers (e.g., demographics, motivation for product use), products (e.g., devices, e-juice, price, discounts), and advertising (e.g., intensity, content). Prior to quantitative observations, vape shop owners/employees were approached and invited to participate in the study, and those who agreed answered a series of open-ended questions (e.g., “How would you describe the customers at your vape shop?” or “For customers who want to quit smoking, what advice do you give?”). The presence of any of several themes expressed in the open-ended responses (e.g., “Noncommittal, try everything, whatever works for you”, “Use a 2nd/3rd generation device”, “Customization by price point”, “Nicotine dose by number of cigarettes/day”) were noted by the trained interviewer. The interviews were supplemented with observational data (e.g., store hours, location of cash register, types of exterior store advertising). Advertisements present in the shops were viewed, and the presence of themes of particular interest (e.g., safety of ENDS, help to quit smoking) was noted, along with a description of the advertisement.

For each question on the observation guide one response per store was recorded. Store employees were asked the prices of different products in order to determine the price range of products (lowest and highest) because prices were not always posted. For each broad category of ENDS device (cigalike/disposables, e-Go or tank style, mods or RBA [rebuildable atomizers]) employees were asked, “What is the price range?” For quantitative closed ended questions (e.g., the highest and lowest price for a 2nd generation device) the prices were recorded for each store, and the mean and standard deviation for each response was calculated across the 23 stores. Employees at each store were also asked clarifying questions (e.g., “How was that discount price calculated?”), because discounting and other sales practices varied by store. Employees were asked, “Do you offer price discounts?” and if they answered yes, were asked, “How often do you offer price discounts?” and “Typically, on average, how much of a % reduction of the original retail price would this be?” These questions did not elicit rich qualitative data amenable to formal qualitative analysis; employees’ responses were recorded by the interviewer (e.g., “Buy 2, get the 3rd half off”), and the response was later classified into a predetermined category (e.g., “Multi-unit discount”). With regards to e-juice, in addition to prices and discounts, employees were asked, “What percent of your revenue stream comes from e-juice sales?” and “How does this compare with the preceding business year’s sales?”

The vape shop assessment tool used in this study was a pilot instrument that was still under development and has not been formally validated. To facilitate improvement of the instrument, all of the open ended responses were systematically reviewed by multiple members of the study team, using a mutually agreed upon coding scheme to classify open ended responses into categories. Any disagreements between members of the team as to how a particular response should be classified were discussed to determine which category best fit that response, or, for example, to consider if a new category should be added to the coding scheme. All data were systematically reviewed and discussed with the aim to elucidate the basis for disagreements, clarify the rationale for coding, or to improve the coding instrument. Because all disagreements were discussed and resolved, formal blind coding and intercoder reliability statistics were not calculated.

## RESULTS

### Store Characteristics

Sampled vape shops were relatively new businesses; the longest running business had been open for 4 years, and the median age of the businesses was 2 years. The shops were small businesses with a median 3 employees per store (range 2–8). Several interviewees reported being unpaid, concerned about store finances or “going out of business”, working for a “friend” or “mentor”, or running a “hobby shop” for the owner’s entertainment. Most vape shops were storefronts in plazas or malls; 2 standalone stores and 1 mall kiosk were included in the sample. A school was visible from the front door of 3 of the vape shops.

### Device Selection

All stores carried second or third generation vape devices, but 83% of stores (19/23) did not carry first generation devices. The stores carried a median of 2 brands of 2nd generation (range 0–4) and a median of 10 brands of 3rd generation devices (range 4–50), respectively. The 2nd generation device brands most frequently named as best sellers were eGo (9 stores), Kanger (5 stores), Aspire (5 stores), Joyetech (3 stores), and Vision (3 stores); the 3rd generation device brands most frequently named as best sellers were Kanger (15 stores), Sigelei (10 stores), Aspire (7 stores), and Joyetech (4 stores).

Aggregated over all 23 sampled stores, the average price of a 2nd generation ego-style e-cigarette ranged between $32 (SD=16) (lowest) and $55 (SD=23) (highest). The average price of a 3rd generation mod-style e-cigarette ranged between $81 (SD=37) (lowest) and $441 (SD=679) (highest). In addition, store employees reported the types of discounts and how frequently they applied discounts to the prices. Almost all stores (96%, 22/23) offered discounts such as percentages off the final price, special offers to loyal customers or students, or holiday specials. Bargaining was also commonplace. The average discount employees estimated for the 22 stores offering discounts was 16% (SD=7) off the retail price. The variety of vape devices and changes in devices over time was also reported to drive sales. Employees posited that rapid technological adaptation, and “habitual mod buyers” who became “addicted” to the technology were responsible for keeping device profits up.

### E-Juice

All stores sold pre-packaged e-juices and about a third (35%) also sold house brands. House brands were made in a variety of ways, including being mixed on site at the store by employees, made in a chemical laboratory with wholesale distribution, or requisitioned from local bulk manufacturers and labeled with the store’s name. Average prices for a typical 15 ml bottle of e-juice ranged from $10 (SD=3) (lowest) to $14 (SD=4) (highest); house brands were comparable with average prices ranging from $10 (SD=3) (lowest) to $11 (SD=3) (highest). Similar to devices, the majority of shops (87%, 20/23) also offered discounts on e-juice, mainly based on quantity (e.g., buy 2, get the 3rd 50% off) and the average discount was 16% (SD=12). Stores sold a wide variety of different e-juice flavors and carried 97 (SD=83) on average.

Nicotine content of the juices ranged from 0–24 mg/ml. Interviewees estimated 6 mg e-juice was most commonly sold, and several commented that newer devices needed less nicotine. Most stores offered free samples of e-juice (91%, 21/23); and the majority restricted the samples to nicotine-free e-juice (57%, 13/23), while the remainder (35%, 8/23) offered free samples containing 1–24 mg of nicotine. When asked to name the three top selling e-juice brands, employees named a total of 40 different brands, 4 stores named their house brand in the top 3 sales (4 stores) and other frequently mentioned brands of e-juice included Cuttwood (10 stores), Suicide Bunny (4 stores), Lost Art (3 stores), OMG (3 stores), and Ruthless (3 stores).

Employees reported customers were interested in new flavors of juice, and one employee mentioned “honestly I think at least every store should be making at least 75% of their profits from juice”. Consistent with this statement, when asked about the percentage of revenue stream coming from e-juice sales, employees estimated that juice was responsible for the majority of store revenue (median 70% of revenue over all 23 sampled stores) and that this number was consistent with the previous years’ revenue (8/14 stores responding to this sub-question).

### ENDS Users

On average, vape shop employees estimated 72% (SD=29) of their customers were interested in smoking cessation or to replace tobacco, and only about 10% (SD=20) for first-time recreational use. However, it was difficult for employees to readily identify if customers were naive to nicotine or not. For example, employees at six shops described a subgroup of customers referred to as “cloud chasers”. This subpopulation of 4–50% of customers were young adult hobbyists “cloud chasing,” with a particular interest in the performance of inhaling and blowing large clouds of aerosol or the technical aspects of vape devices. This group included both current smokers transitioning from tobacco, and new enthusiasts just “discovering nicotine”, or buying devices with nicotine-free juice to fit in with peers.

All of the store employees interviewed reported their customers were “all ages” and 16 stores estimated their customers included young adults in their 20’s. Stores were not asked explicitly about minors, although when asked “Do you know of any local or state restrictions on your shops?” 7 employees (30% of shops) mentioned knowledge of restricted sales to minors. When asked for their opinion on the ideal ENDS regulatory structure, 5 employees (22% of shops) said they did not approve of minors accessing e-cigarettes. Several were skeptical that restrictions on vape shops would prevent minors accessing the products and mentioned that minors were getting e-cigarettes online. A substantial number of stores (41%, 9/23) had posted signs denying entry to minors.

### In-store Advertising and Signs

**Figure 2:**
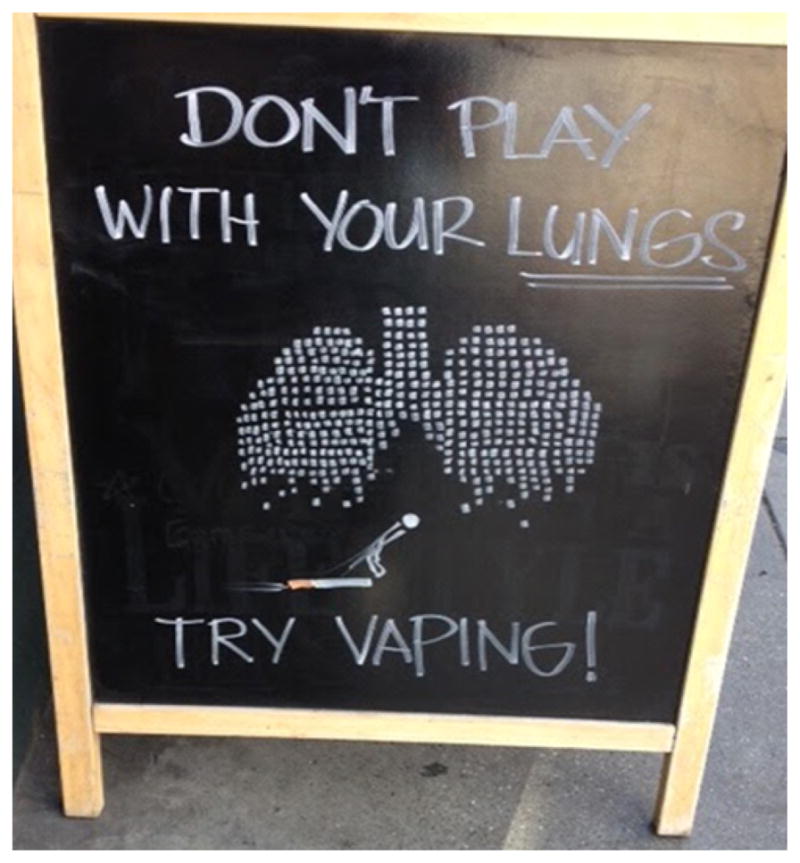
Example of exterior sign indicating vaping is healthier than smoking.

**Figure 3:**
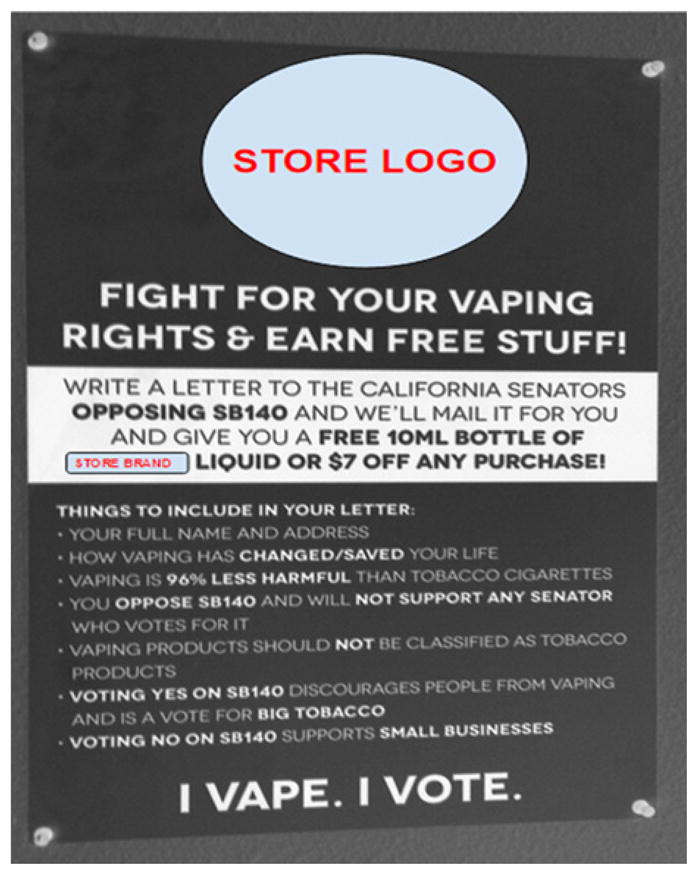
De-identified interior poster offering free e-juice or discount in exchange for customer advocacy actions.

Anti-tobacco industry or anti-smoking attitudes were present on signs in many of the audited vape shops. A third of stores (7/23) had anti-tobacco signage (e.g., “No smoking, try vaping.”) in interior and/or exterior displays. On observation 26% of stores (6/23) displayed a health claim in interior or exterior advertising. The most common claims were for effectiveness of e-cigarettes as smoking cessation aids (26%, 6/23) and the safety of e-cigarettes (9%, 2/23), or indicating that vaping was healthier than smoking (Figure 2). Several stores (22%, 5/23) had vape industry magazines for customers to browse or take home. Some stores had advocacy materials prominently displayed and one store had a poster that offered a “free 10ml bottle of [store brand e-liquid] or $7 off any purchase” in exchange for a letter to a state senator opposing a bill that would classify ENDS as tobacco products (Figure 3).

### Cessation

**Figure 4:**
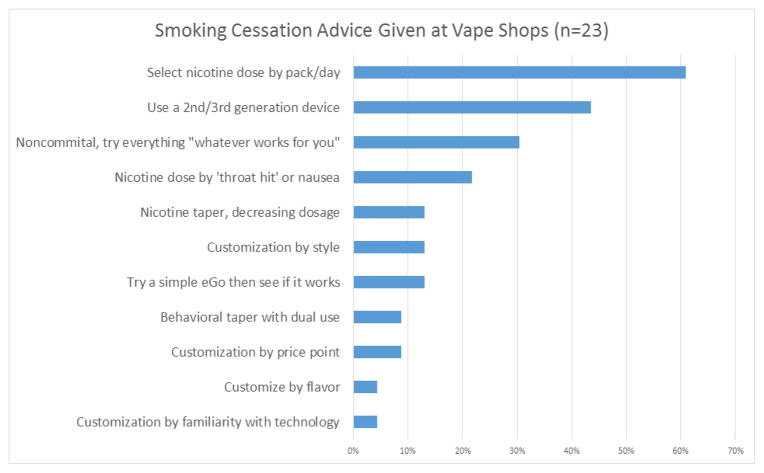
Frequency of types of smoking cessation advice given to customers, as reported by employees of San Francisco/Bay Area vape shops.

All employees interviewed mentioned being asked for or giving smoking cessation advice to customers, although none mentioned training in cessation counseling, and two explicitly mentioned the lack of evidence-based recommendations for using e-cigarettes as a smoking cessation aid. Vape shop employees provided examples of cessation advice they would give in a fictional scenario: “What if I’m a 50 year old 2 pack-a-day smoker? What is your best advice for me to quit?” All 23 store employees interviewed answered this question with some type of cessation advice (Figure 4) and the majority (83%, 19/23) stipulated tailoring a cessation plan to an individual customer or allowing the customers to experiment and tailor for themselves. Employees frequently emphasized that there was no right way to quit and customers should experiment and customize the experience (“whatever works for you”, 30%, 7/23), as the outcome was more important than the method.

The most common advice given was to choose an initial dose of nicotine based on usual cigarette consumption (61%, 14/23). The average starting dose of nicotine recommended for a 2 pack/day smoker was 14 mg (SD=7; range 6–24 mg/ml), decreasing strength over time. For example, one employee reported successful customers typically started with a nicotine dose of 12–18 mg/ml for one month, tapered to 6–3 mg/ml for 3 months, then tapered to 0 mg over three months. Employees at several stores (22%, 5/23) also described adjusting nicotine content of e-juice based on individual response to each device and e-juice combination, which was assessed via customer self reports of “throat hit”, continued cravings, or symptoms of nicotine overdose such as nausea. Nearly half of employees (43%, 10/23) advised use of a 2nd or 3rd generation e-cigarette (rather than a 1st generation “cigalike”) for initial cessation trials.

## DISCUSSION

We found Bay Area vape shops offered a wide variety of vapor devices with substantial opportunities to customize both the device itself and the e-juices. Unlike retail environments that sell tobacco, most vape shop employees surveyed reported that most of their customers were interested in quitting smoking, and all offered smoking cessation advice to customers. While respondents in this study frequently recommended 2nd and 3rd generation devices for smoking cessation, there is little evidence of the efficacy of devices sold in vape shops, although data from other studies suggest that the 2nd and 3rd generation devices have the potential for more effective nicotine delivery^7^.

Vape shop employees in this study also frequently reported their customers were “all ages” or included young adults. This is relevant since other studies have shown that young adults rarely use evidence-based smoking cessation interventions to quit^22,23^. The advice provided by vape shop employees might be viewed similarly to peer support for smoking cessation. While few vape shop employees utilized formal smoking cessation counseling strategies, some study participants reported they worked extensively with heavy smokers for a period of months helping them experiment with different strategies, devices, and e-juices to move towards an outcome (tobacco free) over time. The intensive engagement with customers over time provides social support for quitting and practical advice, both of which are elements of recommended smoking cessation counseling^24^. Vape shops may provide opportunities for smoking cessation through repeated exposure for a large number of smokers, an approach that is consistent with the complex adaptive systems or chaos theory perspective on health behavior change^25^. Given the nature of the business, it is perhaps not surprising that the most frequent cessation advice given to customers in this study focused on device or e-juice characteristics (e.g., selection of nicotine level, 2nd or 3rd generation devices to fit cravings or cigarette consumption). The devices sold in vape shops offered greater options for customization than “cigalikes”, and experimentation with different device or juice options might keep a smoker engaged with the quitting process for a longer period of time. Employees in this study were not formally trained in smoking cessation, although some gave advice that reflected strategies (e.g., setting a quit date, problem solving strategies), which are known to increase success of quit attempts when used with nicotine replacement therapies (NRT)^24^. Further, previous research has shown that vape shop owners generally believe ENDS to be safer than NRT,^15^ so they may be unlikely to recommend it. Those providing training and resources in smoking cessation counseling might consider vape shops as potential partners to improve the quality of advice given and success of quit attempts. However, most trained cessation counselors cannot endorse the use of unregulated devices without proven efficacy for smoking cessation, and vape shop employees may have a financial disincentive to recommend evidence-based alternatives to vaping, such as NRT or medications, to their customers.

The advertisements observed portrayed ENDS as effective therapeutic devices for smoking cessation. This is consistent with previous studies showing that vape shop owners tend to compare ENDS to medical treatments^14^. Limited observational studies suggest that more intensive use of ENDS enhances smoking cessation,^26^ and that 2nd generation devices may be more acceptable to people wishing to quit smoking^27^. Variation in devices and juice manufacture complicates chemical evaluation of ENDS,^28^ however most studies concluded ENDS produce significantly lower levels of tobacco specific nitrosamines and other toxic compounds compared to combustible cigarettes, while 2nd and 3rd generation devices have been found to increase aldehyde^29^ and formaldehyde^30^ production commensurate with higher voltage and temperatures. If, as some employees reported, the vape shops’ profits are driven by ongoing purchases of e-juice or new devices, this might discourage employees from recommending minimizing time using ENDS products, or from encouraging a transition to NRT, as one might do in a clinical setting.

When comparing our findings with the existing literature on vape shops it should be noted that a previous study observing interactions between vape shop customers and employees in Southern California did not report discussions of smoking cessation strategies^17^. On the other hand, other previous studies reported that e-cigarette retailers recommended ENDS for smoking cessation,^31^ or reported having reduced or quit smoking by means of using ENDS^15^. Future studies are needed to determine the extent of smoking cessation advice that is being provided in vape shops in other locations. While the vape shop employees in this study estimated that most of their customers are interested in smoking cessation, they also reported a significant minority of their customers were interested in recreational use. While some of these customers may also be using ENDS as an alternative to cigarettes, for others recreational use may lead to initiation or increase, rather than cessation, of nicotine use.

While not a formal part of this assessment, many shop employees interviewed for this study spontaneously expressed anti-tobacco industry sentiments, and a similar observation was made in a prior study in New Jersey^32^. However, ENDS advocacy groups have mobilized against policies regulating ENDS, including FDA regulation^33^ and one shop in this study used promotional incentives to encourage customers to take action against tobacco control. In theory, vape shops that do not sell other tobacco products might compete with cigarette sellers such as convenience stores and tobacco shops. However, the top ENDS brands are 1st generation cigalike devices largely owned by tobacco companies^34^. It is unclear if vape shops can or would mobilize against the tobacco industry. It is possible that vape shops have greater sales and influence than estimated, as most data on ENDS sales comes from convenience stores (e.g., Nielsen) and independent vape shop sales are harder to track^35^. In addition, ENDS devices sold at vape shops are now included under FDA regulation of tobacco products, and shops that make or modify vaping devices or mix e-liquids may be considered manufacturers under the new deeming rule. The deeming rule went into effect August 8, 2016, and its impact on vape shop businesses and sales practices is not known.

### Research Needs

Researchers and policymakers retain significant concerns regarding ENDS, including the safety of chronic aerosol and nicotine administration, gateway initiation among adolescents and renormalization of smoking, standardization and testing of devices and flavorings, and some respondents in this study anecdotally shared these concerns. Efforts should be made to engage the vape community in collaborative participation in research and to translate results into community practice. Future studies should assess the efficacy of the 2nd and 3rd generation devices sold in vape shops for smoking cessation. Exclusive ENDS users can be difficult to recruit for research, and partnership with vape shops might encourage more of their customers to participate. Efforts to monitor ENDS sales and retail environments should include vape shops. Research assessments of retail vape shops should supplement or precede observational data with in-store interviews, due to the lack of posted prices and frequent bargaining with different pricing for custom devices.

### Limitations

The study has several limitations. While we used prior successful methods to identify vape shops in the Bay Area, we may have missed some shops. Not all vape shops agreed to participate in the study, which may have introduced bias.

The vape shops in the Bay Area were subject to new tobacco control policies and many respondents felt under attack, so those who participated may have been motivated to characterize their customer base in a way that would reflect well on the business. Some businesses declined to participate without assurances that the results of the research would benefit vape shops. The vape shop assessment tool used in this study was a pilot instrument that has yet to be formally validated, so any differences in the interpretation or classification of open-ended responses into categories were discussed by the research team rather than using blind coding and inter-rater reliability statistics.

This study relied on employee estimates of vape shop customer motivations, prices and demographics without objective assessment of the veracity or accuracy of these claims. Surveys of customers and their perceptions and experiences in vape shops should complement the data presented here. While we attempted to reach every vape shop in the Bay Area, the results may not represent vape shops in this or other areas. Lastly, our small sample size forced us to report descriptive results and precluded any hypothesis testing.

## CONCLUSIONS

Vape shops are unique settings with potential to enhance smoking cessation due to the variety and customization of devices and juices available, and their engagement with customers with the stated intent to support smoking cessation. However, most cessation advice focused on product characteristics rather than recommended behavioral counseling practices. Profit motives for vape shops might work against recommending the safest or most efficacious smoking cessation strategies. Future research on vape shops should address the efficacy of the devices and strategies recommended for use, and customer behavior over time. In addition, the impact of FDA regulation of ENDS under the new deeming rule on business practices in vape shops should be assessed. In vape shops, ENDS are frequently characterized by employees as a smoking cessation aid; if the FDA further stipulates under its new regulatory authority that devices sold with therapeutic claims be regulated as drugs or therapeutic devices, it would be logical to regulate ENDS in this context as such.
